# Tanshinone IIA Regulates NRF2/NLRP3 Signal Pathway to Restrain Oxidative Stress and Inflammation in Uric Acid-Induced HK-2 Fibrotic Models

**DOI:** 10.2174/0118715303315786240926075342

**Published:** 2024-10-28

**Authors:** Weiliang Zhang, Jiashu Feng, Ruiqi Liu, Ting Xiang, Xinlin Wu

**Affiliations:** 1 Department of Traditional Chinese Medicine, The First Affiliated Hospital of Sun Yat-Sen University, Zhongshan 2^nd^ Road, Guangzhou, 510080, P. R. China;; 2 Department of Traditional Chinese Medicine, The Second People's Hospital of Shuangliu District, Sixing Street, Chengdu, 610200, P. R. China

**Keywords:** Uric acid, HK-2 fibrosis models, Oxidative stress, Inflammation, Tanshinone IIA, NRF2, NLRP3

## Abstract

**Introduction:**

This study aims to investigate the function and potential mechanism of Tanshinone IIA in uric acid-induced HK-2 fibrosis models.

**Materials and Methods:**

An *in vitro* model of fibrosis was constructed using uric acid stimulation. RT-qPCR and Western blot were used to evaluate the levels of inflammatory cytokines. The detection of ROS and ELISA assay were used to analyze the changes in oxidative stress.

**Results:**

Tanshinone IIA inhibited the increase in inflammatory cytokines TNF-α, IL-1β, IL-6, and IL-18 and the formation of NLRP3 inflammasome induced by uric acid stimulation. In addition, Tanshinone IIA treatment reduced the production of ROS and MDA, promoting the expression of SOD and CAT, thereby protecting HK-2 cells from oxidative stress damage. Besides, the expression of TGF-β, FN, and Collagen I was significantly reduced by the treatment of Tanshinone IIA. Mechanistically, Tanshinone IIA inhibited the expression of inflammatory cytokines and the formation of the NLRP3 inflammasome by targeting NRF2.

**Conclusion:**

Tanshinone IIA exerts a protective role in uric acid-induced HK-2 fibrosis models by targeting the NRF2-NLRP3 signaling pathway to reduce the occurrence of inflammation and oxidative stress.

## 1 INTRODUCTION

Hyperuricemia (HUA), characterized as the fourth hyper-disease, is a perturbation in purine metabolism influenced by both genetic predisposition and environmental factors [1]. Epidemiological studies indicate a global increase in the prevalence of hyperuricemia or gout patients. The prevalence of HUA among Chinese adults is estimated to be approximately 14%, whereas in the United States, it reaches as high as 20% [2, 3]. Currently, the primary approach to alleviating this type of renal injury is to reduce uric acid (UA) levels through the administration of drugs, such as allopurinol and benzbromarone, which inhibit UA production or promote its excretion. However, several studies have confirmed that the above-mentioned drugs have significant toxic side effects, such as hepatotoxicity associated with benzbromarone and allergic reactions induced by allopurinol [4, 5]. Therefore, developing new therapeutic drugs has become an important research direction.

The complications associated with HUA encompass acute kidney injury, chronic kidney disease, and hypertension [6]. Elevated levels of uric acid (UA) can instigate monosodium urate crystals, leading to renal tubular cell death and severe inflammation, ultimately triggering acute kidney injury and renal fibrosis [7, 8]. Furthermore, UA also expedites the progression of chronic kidney disease and hypertension by promoting oxidative stress and inflammatory effects [9-11]. Therefore, meticulous attention should be directed towards the implicated pathways of oxidative stress and immune response mechanisms when addressing HUA [12].

The activation of the NRF2/NLRP3 signaling axis represents a crucial pathway in UA-induced kidney injury. UA has the ability to induce damage to vascular endothelial cells, subsequently leading to mitochondrial structural and functional abnormalities. This process results in the generation of a significant amount of ROS [13]. Moreover, ROS can directly attack large molecules, ultimately leading to cell apoptosis [14]. It follows that oxidative stress induced by ROS may be regarded as a vital pathogenesis of UA-induced kidney disease. The NLRP3 inflammasome is composed of NLRP3, ASC, and Caspase 1, which is a major component of the innate immune system [15]. Research has confirmed that NLRP3 inflammasomes can be activated by UA crystals, exacerbating kidney damage [16]. Meanwhile, there is a study that argued that inhibiting the activation of NLRP3 has a certain effect on the treatment of renal fibrosis, and this function may be related to antioxidants [17]. Therefore, whether it is possible to reform renal fibrosis by inhibiting the generation of ROS and blocking the occurrence of oxidative stress and inflammation has aroused our interest.

Tanshinone IIA (Tan IIA), a significant component of the traditional Chinese medicine Salvia miltiorrhiza (Danshen), is currently utilized primarily for the treatment of cardiovascular diseases [18]. Studies have confirmed that the mechanism of its action is related to antioxidant and anti-inflammatory effects [19]. In renal diseases, studies have confirmed that Tan IIA can protect diabetes nephropathy (DN) by inhibiting oxidative stress and improving cell apoptosis [20]. Our previous experiments argued that Tan IIA could inhibit MCP-1 and IL-1β in UA nephropathy (UAN) rats to exert anti-inflammatory effects [21] and improve mitochondrial status to alleviate UA-induced cell apoptosis [22]. Moreover, mitochondrial damage increased the production of IL-6 and IL-1β, which, in turn, induced fibrosis. Nevertheless, the mechanism is not yet fully explicit. This study aims to investigate whether Tan IIA can improve UA-induced renal fibrosis in HK-2 cells by inhibiting oxidative stress and inflammation and determine the target of its function.

## 2 MATERIALS AND METHODS

### 2.1 Reagents

Tan IIA (S31459-1g) was purchased from Yuanye Biotechnology (Shanghai, China). Antibodies: NLRP3 (13158S), ASC (67824S), Caspase-1 (3866S), Keap1 (8047S), NRF2 (12721S), TNF-α (3707s), IL-1β (12703S), IL-6 (12153S), and IL-18 (67775S) were purchased from Cell Signaling Technology (Shanghai, China). TGF-beta (ab215715, and FN (ab268020) were purchased from Abcam (Shanghai, China). Collagen-1 (GB114197-100) was purchased from Servicebio (Wuhan, China). GAPDH (KC-5G5) was purchased from Kangduan (Shanghai, China). Goat Anti-Rabbit IgG (H+L) and Mouse/Human ads-HRP (4050-05) were purchased from Neobioscience (Shenzhen, China).

### 2.2 Cell Culture

HK-2 cells were purchased from ATCC (Wuhan, China), cultured in DMEM (containing 1% Penicillin-Streptomycin and 10% FBS), and incubated in a CO_2_ incubator with 5% at 37°C.

### 2.3 Cell Grouping and Treatment

HK-2 cells were divided into the normal control group (NC), the UA fibrotic model group (UA), and the Tan IIA treatment group (UA+Tan IIA). Based on previous research results from our research, stable UA-induced HK-2 cell fibrotic models could be prepared by stimulating with 0.5 mg/mL UA for 24 h. UA+Tan IIA was treated with Tan IIA at concentrations of 1, 2, 5, 10, 20, 50, and 100 μM for 48 h after model induction.

In order to verify the changes in indicators of oxidative stress and fibrosis after siRNA, we designed the following 5 groups of experiments: NC, UA, UA+Tan IIA, UA+ Tan IIA +siNC group (siNC), and UA+ Tan IIA +siNRF2 group (siNRF2).

### 2.4 Cell Transfection

siRNA targeting NRF2 and control siRNA were purchased from General Biosystems Limited (Anhui, China). HK-2 cells were seeded in a 6-well culture plate. Cells were transfected with siNRF2 or control siRNA using Lipofectamine 8000 reagent. The cells were treated 12 h after transfection, and their transfection efficiency was validated using Western blot and Quantitative real-time PCR.

### 2.5 CCK-8

HK-2 cells were seeded in a 96-well plate with a cell density of 1×10^4^ cells per well. After treatment and incubation with Tan IIA, CCK-8 reagent (10 μL) was added to each well. After incubation, the absorbance was measured at 450 nm using an enzyme immunoassay analyzer, and the cell viability of each group was calculated. The average value of the OD450 was used to determine cell viability.

### 2.6 ELISA

HK-2 cells were seeded in a 96-well plate with a cell density of 1×10^4^ cells per well. After treatment and incubation, the culture medium of the cells was collected, and the supernatant was used as the sample. The levels of SOD, CAT, and MDA in the supernatant of each group were determined according to the instructions of the ELISA kit.

### 2.7 Measurement of Reactive Oxygen Species (ROS)

After treatment, the cells in a 6-well plate were incubated with a DCFH-DA fluorescent probe (10 μM) at 37°C for 30 mins. The ROS level was observed using a fluorescence microscope, and the fluorescence intensity was calculated using ImageJ.

### 2.8 RT-qPCR

Total RNA was extracted from HK-2 cells using TRIzol reagent (Invitrogen, USA), and then cDNA was synthesized using a reverse transcription kit (Vazyme, Nanjing, China). The RT-PCR reactions were as follows: 40 cycles of 95°C for 15 s, then 60°C for 30 s. GAPDH was used to normalize the transcription of target genes. The primers are listed in Table **1**.

### 2.9 Western Blot

RIPA lysis buffer was added to each group of cells to extract the cellular proteins. The protein concentration of each group was determined using the BCA method. Then, SDS-PAGE and PVDF membrane transfer were performed, with GAPDH as the internal reference. The membrane was incubated with the primary antibody overnight and incubated with the secondary antibody for 1 h the next day. Finally, the proteins were visualized and photographed, and the relative expression levels of each protein were analyzed.

### 2.10 Statistical Analysis

Statistical analysis was performed using GraphPad Prism 9.2.0 software. Differences between the two groups were analyzed using a t-test, and one-way ANOVA was used for comparisons among multiple groups. *p*<0.05 was considered statistically significant. Each experiment was repeated 3 times.

## 3 RESULTS

### 3.1 Determined the Optimal Concentration and Time of Tan IIA.

**Fig. (1):**
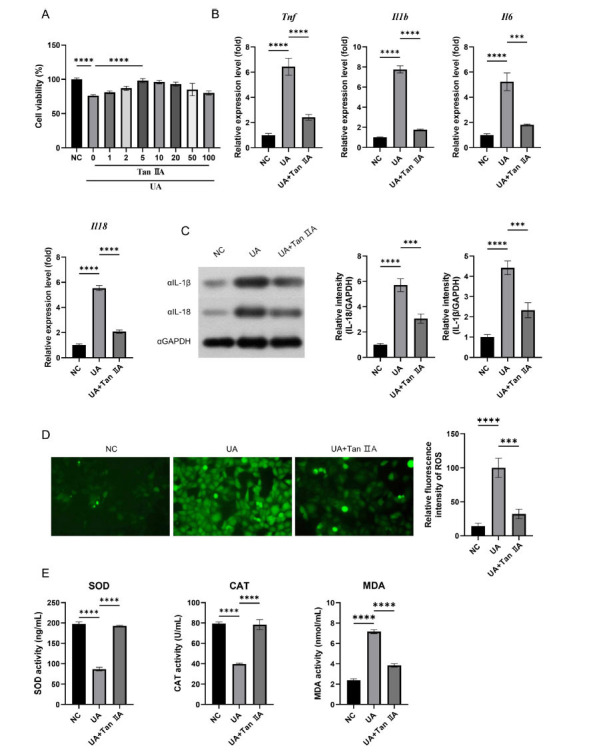
Tan IIA regulated UA-induced inflammation and oxidative stress. The HK-2 cells were cultured for 24 h with or without UA treatment, followed by an additional 48 h culture with or without Tan IIA treatment. **A**: Cell viability was assessed by CCK-8 assay; **B**: The mRNA levels of Tnf, Il1b, Il6, and Il18 were quantified by RT-qPCR analysis; **C**: The protein levels of IL-1β and IL-18 were detected using western blot; **D**: Detection of ROS; **E**: The concentrations of SOD, CAT and MDA were determined by ELISA kits. Values represent the Mean ± SD. *** *p* <0.001; **** *p* <0.0001.

The concentration of Tan IIA was determined by CCK8 assay. UA stimulation at a concentration of 0.5 mg/mL significantly inhibited HK-2 cell proliferation. The results of CCK8 demonstrated that low concentrations of Tan IIA contribute to cellular viability, whereas high concentrations of Tan IIA may exert cytotoxic effects on HK-2 cells (Fig. **1A**). Therefore, for subsequent experiments, Tan IIA at a concentration of 5 μmol/L was selected.

### 3.2 Tan IIA Evaluated Fibrosis by Inhibited Inflammation and Oxidative Stress in UA-stimulated HK-2 Cells

**Fig. (2):**
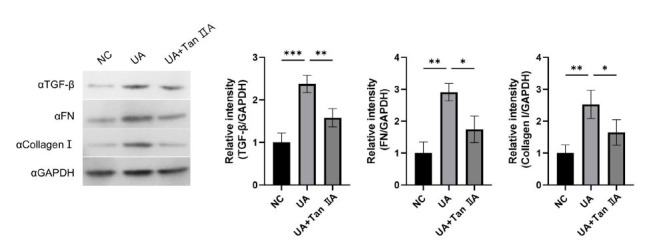
Tan IIA inhibited UA-induced cell fibrosis. The HK-2 cells were cultured for 24 h with or without UA treatment, followed by an additional 48 h culture with or without Tan IIA treatment. Western blot were used to analyze the expression of TGF-β, FN and Collagen I. Data are presented as mean ± SD. *p < 0.05, **p < 0.01, ***p < 0.001.

Oxidative stress is supposed to be a primary causative factor for UA-induced renal diseases. The effects of UA stimulation on inflammatory response and oxidative stress were then examined. The results demonstrated that UA stimulation significantly enhanced the levels of inflammatory cytokines TNF-α, IL-1β, IL-6, and IL-18, whereas Tan IIA inhibited the increase in the expression of these inflammatory factors (Figs. **1B** and **C**). Furthermore, oxidative indicators (ROS, SOD, CAT, and MDA) were evaluated. Tan IIA reversed the increasing levels of ROS and MDA and the decreasing levels of SOD and CAT mediated by UA (Figs. **1D** and **E**). The relationship between inflammation and fibrosis is closely associated. To further investigate whether this response induces fibrosis, we detected fibrosis-related proteins, including TGF-β, FN, and Collagen I. Concurrently, the results showed that Tan IIA treatment has a significant decrease in fibrosis levels, which also suggests that the anti-fibrosis effect of Tan IIA may be through the intervention of anti-inflammatory and anti-oxidative agents (Fig. **2**). Therefore, Tan IIA exerted a protective effect on UA-stimulated HK-2 cells.

### 3.3 Tan IIA Attenuated UA-Induced Inflammatory Response and Oxidative Stress by Targeting NRF2-NLRP3 Signal Axis

**Fig. (3):**
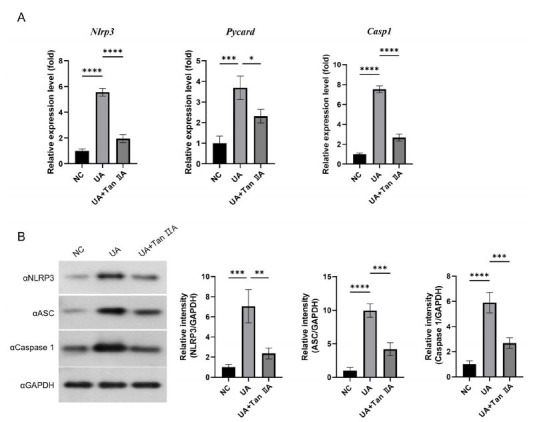
Tan IIA inhibited NLRP3 inflammasome. The HK-2 cells were cultured for 24 h with or without UA treatment, followed by an additional 48 h culture with or without Tan IIA treatment. **A** and **B**: RT-qPCR and western blot were performed to assessed the expression of NLRP3, ASC and Caspase 1. Values represent the Mean ± SD. * *p* <0.05; ** *p* <0.01; *** *p* <0.001; **** *p* <0.0001.

A previous study demonstrated that the NLRP3 inflammasome is involved in the inflammation of HUA. Therefore, we investigated whether Tan IIA targets NLRP3 inflammasome to exert its function. Our results showed that Tan IIA significantly suppressed the mRNA and protein expressions of NLRP3, ASC, and Caspase 1 compared to the UA group (Figs. **3A** and **B**). These results suggested that Tan IIA can inhibit the NLRP3 inflammasome to attenuate the UA-induced inflammatory response.

### 3.4 The Antioxidant and Anti-Inflammatory Activities Of Tan IIA Were Dependent on NRF2 in UA-Induced HL-2 Fibrotic Models

**Fig. (4):**
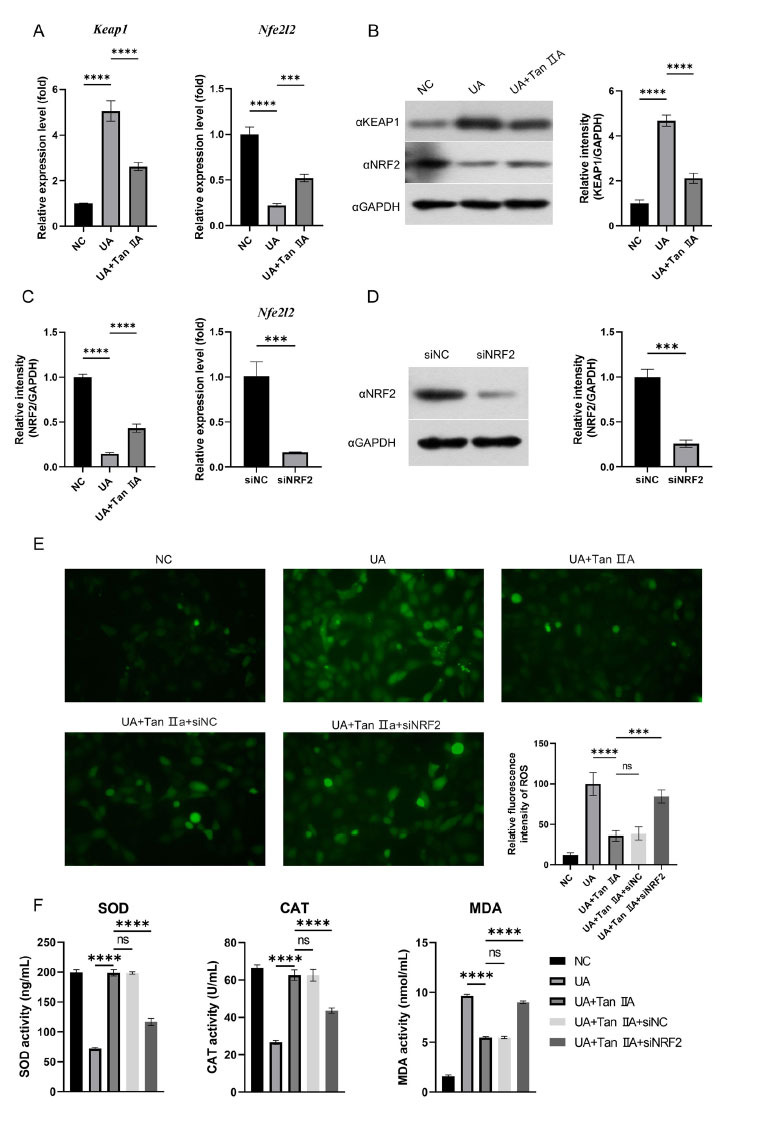
Tan IIA inhibited UA-induced oxidative stress by targeting NRF2. (**A**) and (**B**): The HK-2 cells were cultured for 24 h with or without UA treatment, followed by an additional 48 h culture with or without Tan IIA treatment. KEAP1 and NRF2 expression were evaluated by RTqPCR (A) and western blot (B); C-F: The cells were transfected with siNRF2 or siNC for 12 h prior to stimulation with or without UA and Tan IIA. (**C**) and (**D**): Efficiencies of NRF2 knockdown in HK-2 cells; (**E**): ROS detection; (**F**): The concentrations of SOD, CAT and MDA were determined by ELISA kits. Values represent the Mean ± SD. *** *p* <0.001; **** *p* <0.0001.

Previous studies demonstrated the critical role of NRF2 signaling and ROS production in activating the NLRP3 inflammasome. To investigate whether Tan IIA regulates oxidative stress through NRF2 signaling, the expression of KEAP1 and NRF2 after UA stimulation was detected. UA inhibited NRF2 production by activating KEAP1 expression, while Tan IIA decreased KEAP1 expression and restored NRF2 production, indicating that Tan IIA regulated UA-induced inflammatory response through NRF2-NLRP3 signaling pathway (Figs. **4A** and **B**). Subsequently, NRF2 was knocked down using siRNA, and the results of knockdown, including WB and RT-qPCR, are shown in Figs. (**4C** and **D**). The indicators of oxidative stress were detected, and results revealed that Tan IIA treatment did not significantly reduce ROS production in the case of NRF2 knockdown (Fig. **4E**). Furthermore, the knockdown of NRF2 also inhibited the increased levels of SOD and CAT and promoted the decreasing levels of MDA mediated by Tan IIA (Fig. **4F**).

**Fig. (5):**
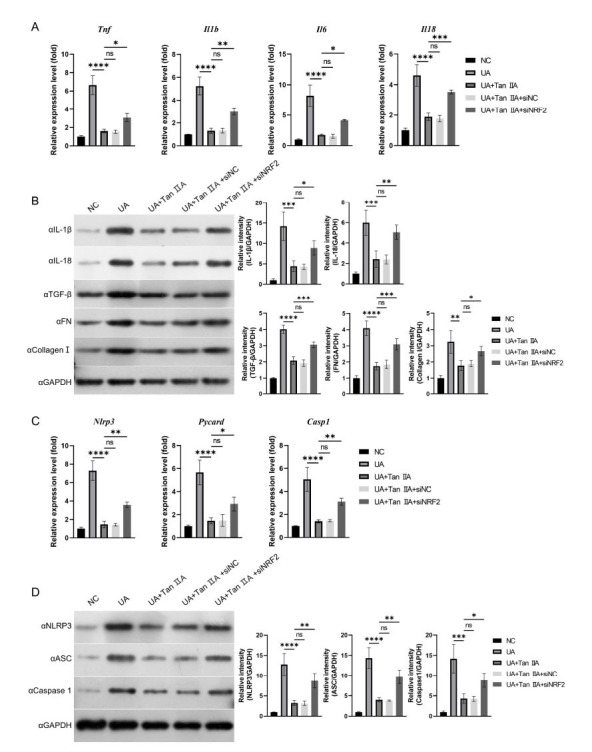
Tan IIA inhibited UA-induced inflammation and cell fibrosis by targeting NRF2. The cells were transfected with or without siNRF2 or siNC for 12 h prior to stimulation with or without UA and Tan IIA. **A** and **B**: RT-qPCR and western blot were performed to assessed the expression of TNF-α, IL-1β, IL-6, IL-18, TGF-β, FN and Collagen I. **C** and **D**: RT-qPCR and western blot were performed to assessed the expression of NLRP3, ASC, Caspase 1. Values represent the Mean ± SD. * *p* <0.05; **p <0.01; *** *p* <0.001; **** *p* <0.0001.

Subsequently, we examined the anti-inflammatory and anti-fibrosis effects of Tan IIA in NRF2 knockdown HK-2 cells. A similar response was observed in the expression of TNF-α, IL-1β, IL-6, and IL-18, highlighting the original regulatory role of TGF-β, FN, and Collagen I (Figs. **5A** and **B**). We further tested the expression of NRF2/NLRP3 pathway-related proteins, and the results showed that the regulatory effect of Tan IIA in this signal axis failed (Figs. **5C** and **D**). These results suggested that siNRF2 abolished the antioxidant, anti-inflammatory, and anti-fibrosis effects of Tan IIA, indicating that the protective effect of Tan IIA is dependent on NRF2.

## 4 DISCUSSION

UA is a crucial metabolic substance in the human body, and its metabolic disorders can contribute to various metabolic diseases. Although the close association between UA and renal damage disease is well-established, the precise mechanism by which elevated UA levels lead to kidney damage remains incompletely understood [23]. Clinical studies have indicated that high UA levels play a significant role in promoting kidney damage [24]. Danshen, an herbal medicine with a history of over two thousand years in East Asia, is traditionally believed to possess bitter and cold properties that enhance blood circulation and resolve stasis [25]. The hydrophilic and lipophilic components of Danshen, such as Tan IIA, are currently widely utilized for treating cardiovascular and cerebrovascular diseases [18]. Our experimental findings demonstrated that Tan IIA exhibits notable efficacy in ameliorating high UA-induced fibrotic damage in HK-2 cells.

The dual effects of UA include both antioxidant and prooxidant activities, with the former primarily exerted extracellularly [12]. However, upon cellular entry, UA rapidly induces oxidative stress by activating NADPH and subsequently promoting ROS production and release, ultimately accelerating cell apoptosis [26]. Oxidative stress is a non-traditional risk factor for various kidney diseases, wherein ROS plays a crucial role in its pathogenesis by forming a vicious cycle [27]. Maintaining intracellular homeostasis relies on the regulation of ROS levels, as different levels can elicit distinct cellular responses [28]. Under normal conditions, ROS plays an important role in activating signaling pathways, and cell viability depends on the basal level of ROS [29]. Nevertheless, excessive generation of ROS induced by UA disrupts the balance between oxidation and antioxidant activity within the body due to surpassing physiological clearance mechanisms [30, 31].

NRF2, a transcription factor pivotal in maintaining redox homeostasis, exerts its protective effects by upregulating the expression of genes encoding antioxidant enzymes [32]. Given its demonstrated efficacy in both acute kidney injury and chronic kidney diseases, NRF2 emerges as a promising therapeutic target for renal disorders [33-36]. In our experiment, Tan IIA cleared excess ROS by increasing the expression of NRF2, which is consistent with the findings of other studies. SOD and CAT are important factors that constitute the line of defense against oxidative stress, and MDA is one of the main markers of oxidative stress [30, 37]. Our study demonstrated that Tan IIA not only restored decreased SOD and CAT caused by UA but also reduced MDA levels *via* modulation of NRF2 activity. These results further confirmed the potent antioxidant effect exerted by Tan IIA.

Studies have reported that high levels of ROS can activate NLRP3 inflammasomes [38, 39]. (+)-Catechin enhances the NRF2 antioxidant pathway to suppress ROS-induced activation of the NLRP3 inflammasome [40]. The activation of the mitochondrial ROS-NLRP3 inflammasome pathway in chronic kidney disease is a pivotal factor contributing to the apoptosis of renal tubular epithelial cells, while NRF2 exerts an antioxidant and anti-inflammatory role by attenuating mitochondrial ROS-mediated NLRP3 inflammasome activation, thereby mitigating high-fat-induced kidney injury [41]. UA mediates cell apoptosis through oxidative stress and inflammation, and intervention-related factors can effectively inhibit oxidative stress-induced kidney damage [13]. NLRP3 inflammasome could promote the production of inflammatory factors IL-1β and IL-18 [42]. IL-1β and IL-18 induce the production of various factors of inflammatory and fibrotic, like TNF-α and TGF-β [43]. TGF-β is a key driver of tissue fibrosis [44]. In addition, ROS can also release pro-inflammatory factors, TNF-α and IL-6, by regulating other pathways [38]. Studies have suggested that salvianolic acid b, another component of Danshen, can inhibit the effect of NLRP3 inflammasomes by enhancing the expression of NRF2 [45]. We found that intervention of UA led to an increase in the expression of NLRP3, ASC, and Caspase-1 proteins in HK-2 cells, and the levels of inflammatory factors, such as IL-1, IL-6, IL-18, TNF-α, *etc.,* were significantly enhanced. At the same time, the level of fibrosis was also promoted clearly, indicating that oxidative stress and inflammation are important risk factors for UA-induced renal fibrosis. However, these injuries were significantly reversed by Tan IIA treatment. This also suggests that the anti-fibrosis effect of Tan IIA may be achieved through antioxidant stress and anti-inflammatory, which means that Tan IIA exerts antioxidant stress and anti-inflammatory effects in UA-induced HK-2 cell fibrotic model by regulating NRF2/NLRP3 activation. To validate this idea further, we knocked down NRF2 within cells, and the results showed that the knockdown of NRF2 eliminated the protective effect of Tan IIA in UA-induced renal fibrosis. Meanwhile, Tan IIA treatment was no longer able to effectively exert its original antioxidant stress and anti-inflammatory effect. More relevantly, Tan IIA treatment failed to inhibit the expression of fibrotic factors, highlighting that the function of Tan IIA in regulating NRF2/NLRP3 for treating UA-induced renal fibrosis depends on NRF2 activation.

It is worth noting that although Tan IIA has been confirmed as a drug with anti-inflammatory, antioxidant, and neuroprotective properties, it is advisable to be vigilant about the dosage of Tan IIA. Research has confirmed that Tan IIA exerts a protective effect on H9C2 cell without any cytotoxicity (less than 10 μM) [46]. Moreover, some studies also suggest that high concentrations of Tan IIA exhibited toxicity and growth inhibition effects on zebrafish embryos, but there was no significant change in the inhibitory effect at concentrations less than 6 μM [47]. As shown in Fig. (**1**), different concentrations of Tan IIA were used to treat the UA-induced HK-2 cell fibrotic model, and optimal concentration was confirmed at 5 μM. This result is consistent with the concentration determined in other studies. In addition, our study is limited to *in vitro* experiments and cannot accurately simulate the metabolism of Tan IIA *in vivo*. Therefore, its functions in *in vivo* settings remain unclear. Nevertheless, elucidating this matter will be a primary focus of our forthcoming research.

## CONCLUSION

In summary, our data manifested that Tan IIA exerted a significant protective effect on UA-induced renal fibrosis. Mechanically, Tan IIA exerted antioxidant stress and anti-inflammatory effects in UA-induced HK-2 cell fibrotic models by regulating NRF2/NLRP3 activation. Simultaneously, the protective effect was offset after NRF2 knockdown, indicating that the anti-fibrosis effect was NRF2 dependent. Whether Tan IIA has a regulatory effect on UA levels depends on further improving relevant theories in animal experiments.

## Figures and Tables

**Table 1:** Sequence of primers for real-time PCR.

Primer	Forward Sequence (5’ to 3’)	Reverse Sequence (5’ to 3’)
TNF-α	TGCTGGCAACCACTAAGAAT	GGCCTAAGGTCCACTTGTGT
IL-1β	TCTCTCCTTTCAGGGCCAAT	ATGTGGCCGTGGTTTCTGT
IL-6	CAATGAGGAGACTTGCCTGG	TGGACTGCAGGAACTCCTTA
IL-18	CATTGACCAAGGAAATCGGC	TCATGTCCTGGGACACTTCT
NLRP3	GCTTCGACATCTCCTTGGT	CCAGAGCTTCTTCAGATTGC
ASC	TTATCGCGAGGGTCACAAAC	GGCTGGTGTGAAACTGAAGA
Caspase-1	TTTGAGCAGCCAGATGGTA	CCTGGGAAGAGGTAGAAACA
KEAP1	GCTGTCCTCAATCGTCTCCTT	ATAGCCCCCAGCAGCATAGAT
NRF2	ATGATGCCCAATGTGAGAAC	TCTACAGGGAATGGGATATGG
GAPDH	GGGAAACTGTGGCGTGAT	GAGTGGGTGTCGCTGTTGA

## Data Availability

All data generated or analysed during this study are included in this published article.

## LIST OF ABBREVIATIONS

Tan IIA: Tanshinone IIA
DN: Diabetes Nephropathy
ROS: Reactive Oxygen Species
UAN: UA Nephropathy
HUA: Hyperuricemia
UA: Uric Acid
